# Performance analysis of the salinity based on hexagonal two-dimensional photonic crystal: computational study

**DOI:** 10.1038/s41598-022-25608-1

**Published:** 2022-12-22

**Authors:** Hassan Sayed, M. Al-Dossari, Mohamed A. Ismail, N. S. Abd El-Gawaad, Arafa H. Aly

**Affiliations:** 1 TH-PPM Group, Physics Department, Faculty of Sciences, Beni-Suef University, Beni Suef, 62514 Egypt; 2 Department of Physics, Faculty of Science, King Khalid University, Abha, 62529 Saudi Arabia; 3 Faculty of Technology and Education, Beni-Suef University, Beni Suef, 62521 Egypt; 4 Physics Department, University College in Al-Aarda, Jazan University, Jazan, 82817 Saudi Arabia; 5 Faculty of Sciences, King Khalid University, Mohayel Asser, Abha, 61421 Saudi Arabia

**Keywords:** Materials science, Optics and photonics

## Abstract

We have designed a unique structure for a liquid sensor based on two-dimensional PCs with a triangular lattice constant in the periodicity by drilling a hexagonal cylinder in a dielectric host material. Using the COMSOL multiphysics approach, we investigated the given structure and sensing performance based on the finite element method. We will optimize two-dimensional hexagonal photonic crystals to localize the photonic band gap region in the mid and far infra-red frequency range, as water is a good absorber for this range of frequencies. Then, we inject the central hexagonal cylinder with saline water and calculate the sensor parameters for different values of the refractive index of saline water at different frequencies related to photonic band gaps. We could reach the optimum conditions of the salinity sensor as the half diagonal of the hexagonal shape (R) = 500 nm, the perpendicular distance between the two diagonal hexagonal (D) = 250 nm, and the number of periods (N) = 5, which gives a high efficiency with sensitivity (S) = 525 nm/RIU, figure of merit (FOM) = 80.7 RIU^−1^, and quality factor (Q) = 375. The effects of structural characteristics on sensing performance are investigated, with new approaches for improving salinity sensors proposed**.** Furthermore, traditional salinity sensors may be replaced by the proposed method in the photo-sensing application, which is simple and practical for use in the thermal desalination techniques.

## Introduction

Photonic crystals were regarded as new materials structures with optical properties that are periodically modified^1–5^. PCs are currently a prominent technology in photonics methods^6,7^. Because their optical constants depend on the incident wavelength, they are also considered a dispersive medium. PCs have attracted a lot of interest due to their unusual interaction with EMW^8^. In addition, PCs can control and regulate the transmission of EMW. So that the EMW can be focused on an active region as per the requirements of the application^9^. The photonic band gap (PBG) is a significant aspect of PCs, wherein, the electromagnetic modes are unable to propagate through the structure in this region of incident wavelengths. Thus, these PBGs have a forbidden zone for photon propagation, but they nonetheless allow for the presence of localized modes and restricted optical waves^10^. This property opens a great tendency in light management to solve the power dissipation in optical applications. Therefore, it can also be employed in a variety of fields, such as photo detection^11^ and sensing^12^. Otherwise, solar energy conversion^13–15^ and PC-based water desalination^16^ are on the rise.

Scientists and researchers have recently focused their efforts on the examination of desalination technologies. Hence, the salinity sensor is very important to determine the salinity level of the generated freshwater. The salinity (S) defined as the quantity of salt in gram dissolved in 1gm of saltwater and represented in parts per thousand (PPT), indicates the amount of salt in seawater. The open ocean's salinities have been observed to be between 34 and 37 PPT, which may also be expressed as 34 to 37 practical salinity units (PSU). Wherein, seawater with *S* equal to 35 contains approximately 35 g of salt and 965 g of water, or 35 ppt (35 PSU). Hence, the water could be used for irrigation and human consumption at $S\le 0.5 \left(PPT\right)$^17^. The interaction of EMW and saline water is the fundamental method of salinity level detection that has been used for decades^18,19^. Hence, PCs—the interaction of EMW with matter—has recently been widely employed in identifying fluid applications as they may provide high degrees of sensitivity for changing the refractive index. Also, according to the formula of thermo-optical effect, PCs can be utilized as temperature sensors because the refractive index varies with temperature^20^. Therefore, PCs could be used to determine the refractive index of liquids and gases according to previously published research^21–23^. As a result, employing PCs technology is a new class of desalination technology, especially in determining the salinity level^24^. The sensor precept is depending on the generation of defect modes within PBG ranges which are due to the variations in the refractive index of the surrounding fluid. Biological analytes can also be detected with two-dimensional PC micro-cavity sensors and theoretical considerations^25^.

Researchers are interested in two-dimensional PCs because they can be utilized as waveguides and light controls^26^. Furthermore, the triangular lattice of two-dimensional PCs is used to design ultra-compact logic gates^27^. Also, As2Se3-chalcogenide with triangle lattice rods within air are used to design digital logic gates and all-optical power splitters by building a line defect (removing rods) in the PCs diagonal direction^28^. Because of the popularity of 2D-PCs, we tend to design a salinity sensor based on the fundamental properties of 2D-PCs with a triangular lattice constant and a hexagonal unit cell, as we will discuss in detail in the following section.

The purpose of this work is to show how 2D–PCs with a triangular lattice constant and the unit cell being hexagonal can determine the salinity level of seawater. The salinity is represented by the saline water refractive index, which are change from 1.3326 to 1.3505 RIU. We have investigated the normal transmission through a slab perforated with hexagonal holes at triangular intervals. Then, the sensor's performance will be evaluated by calculating a variety of parameters such as figure of merit (FOM) quality factor (Q) and sensitivity (S)^29,30^.

## Modeling and simulation

**Figure 1:**
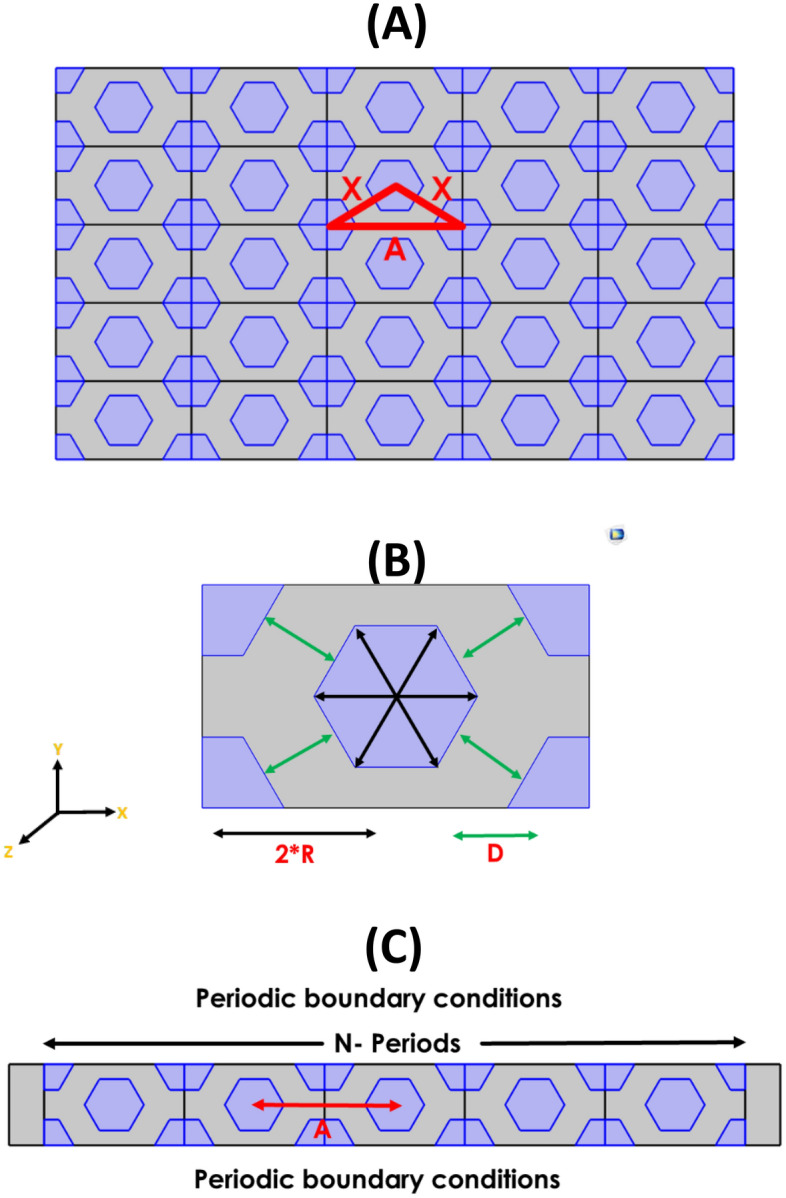
Schematic structure of 2D-PCs with hexagonal unit cell (**A**) surface of the structure with the distances between the hexagonal as shown, (**B**) one unit cell, and (**C**) the array of the unit cell in x direction.

We look into the present structure theoretical modeling. The finite element method[FEM] is the fundamental mathematical method used in the COMSOL multiphysics simulation procedure^31,31^. As we have shown in Fig. 1, the considered structure is comprised of hexagonal drilled holes that unite cells inside a host material matrix with the triangular array in two directions and homogenous in the third direction, which we believe to be two-dimensional PCs.

In Fig. 1, X is the diagonal lattice parameter (center to center), R is the half diagonal of the hexagonal shape, and D is the perpendicular distance between the two diagonal hexagonal as shown. A is the lattice parameter in the X direction as shown, wherein

1 $$x=D+\left(2 R\mathrm{sin}\left(60\right)\right)$$

2 $$A=2 \sqrt{{x}^{2}+{\left(\frac{D}{2}+R\mathrm{sin}(60)\right)}^{2}}$$

The considered structure in Fig. 1 has more than one parameter, such as lattice parameter (center to center), R, the half diagonal of the hexagonal shape, and D, the perpendicular distance between two diagonal hexagons as shown. Therefore, we study the optical properties of the considered structures as shown in Fig. 1 to get the high sensation property. We can see in Eq. (3), the index of water is dependent on the different parameters: salinity S (%), seawater temperature (°C), and wavelength ($\lambda$) in nm^32,33^.

3 $$n\left(S,T,\lambda \right)=1.314+\left(1.779\times {10}^{-4}-1.05\times {10}^{-6}T+1.6\times {10}^{-8}{T}^{2}\right)S-2.02\times {10}^{-6}{T}^{2}+\left(\frac{15.868+0.01155S-0.00423T}{\lambda }\right)-\left(\frac{4382}{{\lambda }^{2}}\right)+\left(\frac{1.1455\times {10}^{-6}}{{\lambda }^{3}}\right)$$

For the considered structure in Fig. 1, it consists of drilled hexagonal holes in a host material of titanium dioxide. All holes are occupied with air, so it is expected that the central hole, which is filled with saline water, will be detected. Finally, the performance of the considered salinity sensor is computed by several factors as we have mentioned. The Eqs. (4, 5, and 6) below are commonly used to calculate these values^34^.

4 $$S=\frac{\Delta \lambda }{\Delta n}$$

5 $$Q=\frac{{\lambda }_{r}}{FWHM}$$

6 $$FOM=\frac{S}{FWHM}$$

where $\Delta \lambda$, $\Delta n$ and ${\lambda }_{r}$ are the wavelength differences, refractive index change, and central wavelength, respectively. FWHM represents the full wave**s** at half-maximum.

Our simulation procedure is performed in two dimensions, with homogenous properties in the third dimension. The boundary conditions of the simulation procedure for the 2D-PCs with a hexagonal unit cell of saline water are periodic conditions for the two sides perpendicular to the wave propagation direction as in Fig. 1C. Also, the mesh size must be 10 times smaller than the smallest incident wavelength with a free triangular size to get more accurate results in the finite element method. Therefore, the simulation meshing parameters are the maximum element size equal to $70 (\mathrm{nm})$, minimum element size equal to 0.213 $(\mathrm{nm})$, and the maximum element growth rate is 1.1. We substitute the refractive index of the host material $(Ti{O}_{2})$ to be 2.5^35^ in the defining materials part of the model.

## Results and discussions

In this part, we will present the theoretical results and discussions for the optimization procedure of our structure to be highly sensitive to any change in the index (n) of a saline water which are depend on the salinity. The results and discussions presented here are presented in two stages: first, we will optimize 2D-hexagonal PCs to localize the photonic band gap region at a specific frequency range related to the optical properties of seawater. Then, through the second stage, we are concerned with the defected two-dimensional photonic crystals, wherein the defect layer of the structure is the saline water. Finally, we could achieve the optimum condition of the salinity sensor furthermore in the photo-sensing application.

### Photonic band gap optimization

**Figure 2:**
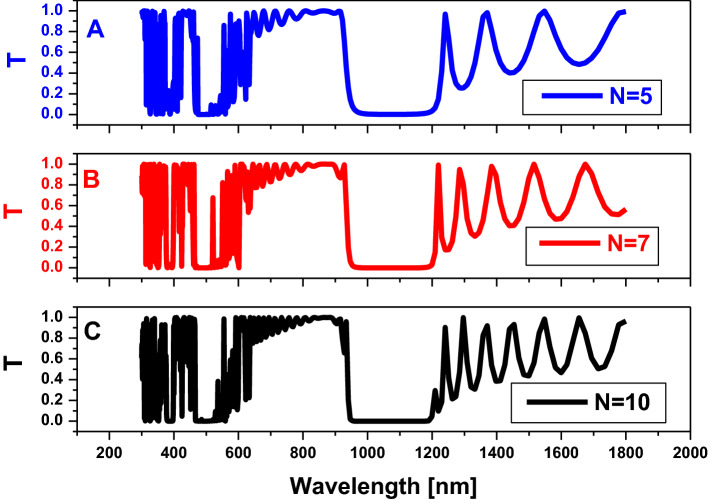
Transmission property of a two-dimensional hexagonal cylinder unit cell with, $R=80 nm$,$D=160 nm,$ and all cylinders being filled with air at the host material of titanium dioxide $(Ti{O}_{2})$ with variations of number of periods as shown.

Here, we study the effect of each parameter, such as the number of periods (N), the half diagonal of the hexagonal shape (R), and the perpendicular distance between two diagonal hexagonal cylinders (D), on the optical properties of the considered structure of 2D-hexagonal-PCs, especially on PBG width and position. Thus, studying the effect of the number of periods is shown in Fig. 2. Figure 2 represents the transmission spectrum of the two-dimensional hexagonal unit cell. All cylinders (hexagonal) with a radius $( R)=80 \mathrm{nm}$, are filled with air, and $D=160 \mathrm{nm},$ at host material from titanium dioxide $(Ti{O}_{2})$ for different values of N as shown. Here the PBG position is almost constant by increasing the value of N in the range of wavelength roughly from 940 to 1200 nm as has been shown, but it causes the sharpness of the PBG edges.

**Figure 3:**
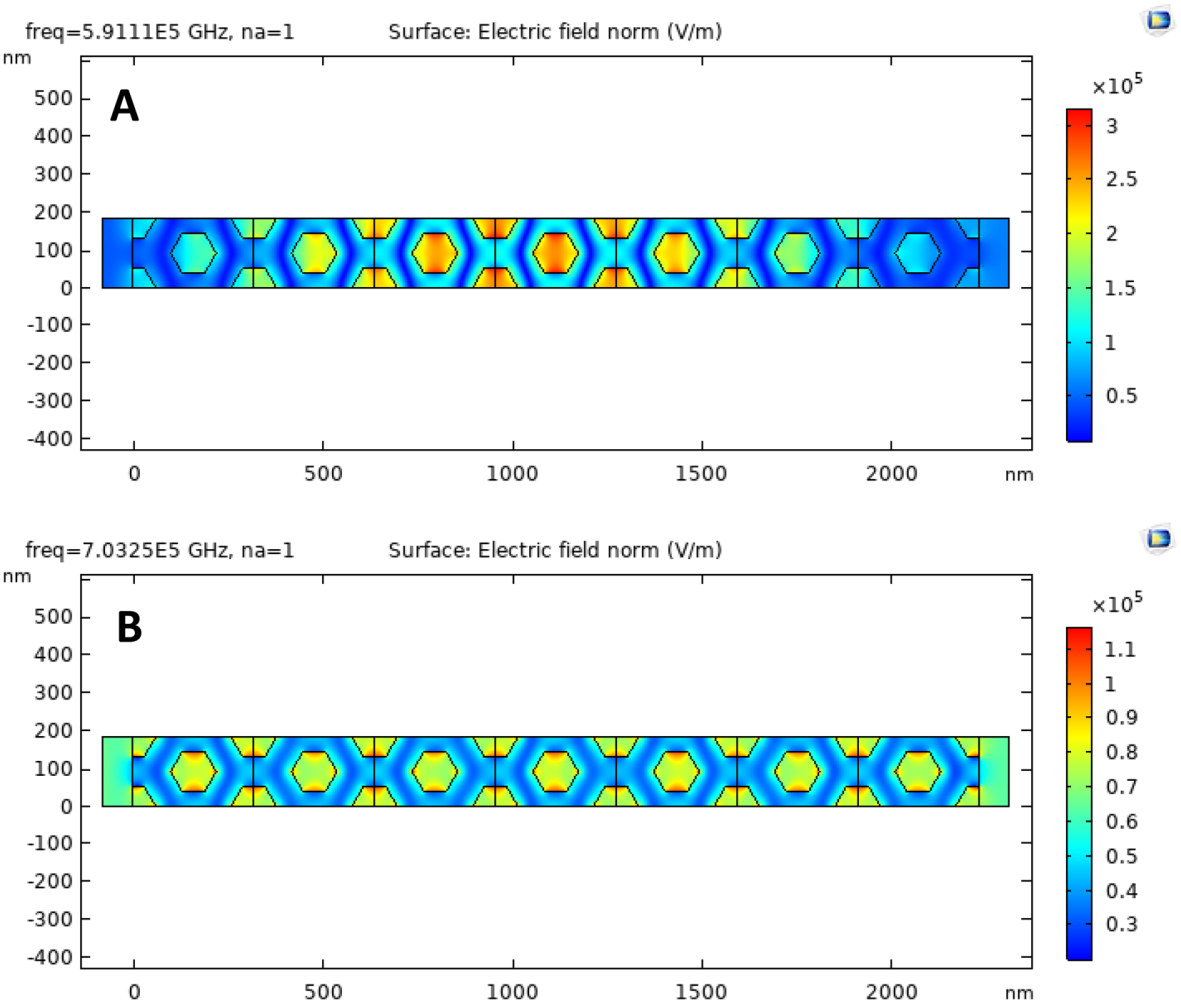
The electric field distribution inside the structure of a two-dimensional hexagonal cylinder unit cell with $R=80 \mathrm{nm}$, $D=160 \mathrm{nm},$ and all cylinders are filled with air at the host material of titanium dioxide $(Ti{O}_{2})$. Also, N periods equal 7 for different values of incident frequency as in (**A**) and (**B**).

Furthermore, we discovered that, for higher values of N periods, the resonance peaks are sharper than the others. Therefore, we optimize to produce the complete structure for this higher number of periods to enhance our sensor's sensitivity. Figure 3 illustrates the distribution of electric fields through the considered structure. It also localize the electric field within pours of the presented structures, which gives the structure the advantage of distinguishing between the different refractive indices.

**Figure 4:**
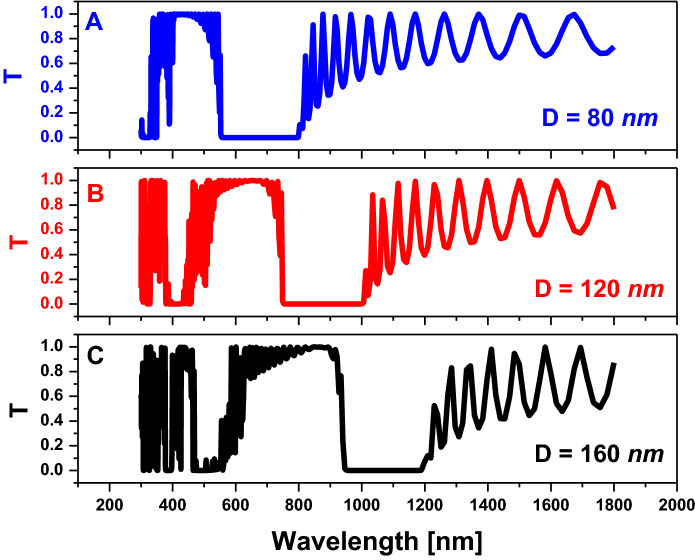
Transmission property of a two-dimensional hexagonal cylinder unit cell with $R=80 \mathrm{nm}$, $N=11,$ and all cylinders are filled with air at host material of titanium dioxide $(Ti{O}_{2})$ at different values of $D$ as shown.

In Fig. 3, we take one array of the hexagonal unit cells with a perfect matching layer in the direction of wave propagation and a periodic boundary condition in the normal direction. We also studied the distribution of the electric field inside the structure, as we have shown. Therefore, at this certain frequency, as we have shown in Fig. 3a, we noticed that the localization of the electric field on the central pore is altered. Therefore, we use this frequency to differentiate between different analytes in this pore. Then, we optimize the value of the perpendicular distance between two hexagonal pores $(D)$ for localization of the PBG in a specific region as shown in Fig. 4. In Fig. 4, by increasing the value of the resonance peaks, they become sharper than the others and the PBG region is shifted towards the longer wavelengths.

**Figure 5:**
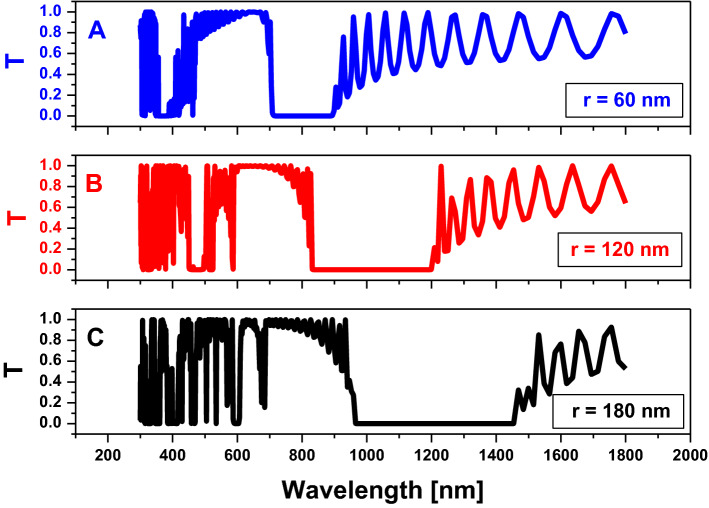
Transmission property of the two-dimensional hexagonal cylinder unit cell with $\mathrm{D}=120\mathrm{ nm}$,$\mathrm{N}=11,$ and all cylinders are filled with air at host material from titanium dioxide $({\mathrm{TiO}}_{2})$ at different values of $\mathrm{r}$ as shown.

Finally, we optimize the value of *R,* which is considered as the half diagonal of the hexagonal shape, as we discussed previously in Sect. 2. The objective of the optimization is the localization of the PBG in the near or mid-infra-red spectrum owing to the optical properties of water. In Fig. 5, by increasing the value of $R$, the PBG width is increased and the position of the PBG is shifted to longer wavelengths as we have shown. Therefore, we collect the last results in Table 1, which represent the optimization process of PBG in the specific frequency range that we need for our application of salinity sensor. From Table 1, to localize a wide PBG in the IR spectrum, we must choose the structure with higher values of each R and D.

**Table 1:** The parameters of each figure with the width and position of the PBG.

Figure	Parameters			Photonic Band Gap		
	$R [\mathrm{nm}]$	$D [\mathrm{nm}]$	$N$	Position		Width $[\mathrm{nm}]$
				From $[\mathrm{nm}]$	To $[\mathrm{nm}]$	
Figure 2	$80$	$160$	5	950	1206	256
	$80$	$160$	7	942	1208	266
	$80$	$160$	10	938	1202	268
Figure 4	$80$	80	11	547	789	242
	$80$	120	11	749	1004	255
	$80$	180	11	938	1194	256
Figure 5	$60$	$120$	11	710	894	184
	$120$	$120$	11	830	1206	376
	$180$	$120$	11	965	1457	492

**Figure 6:**
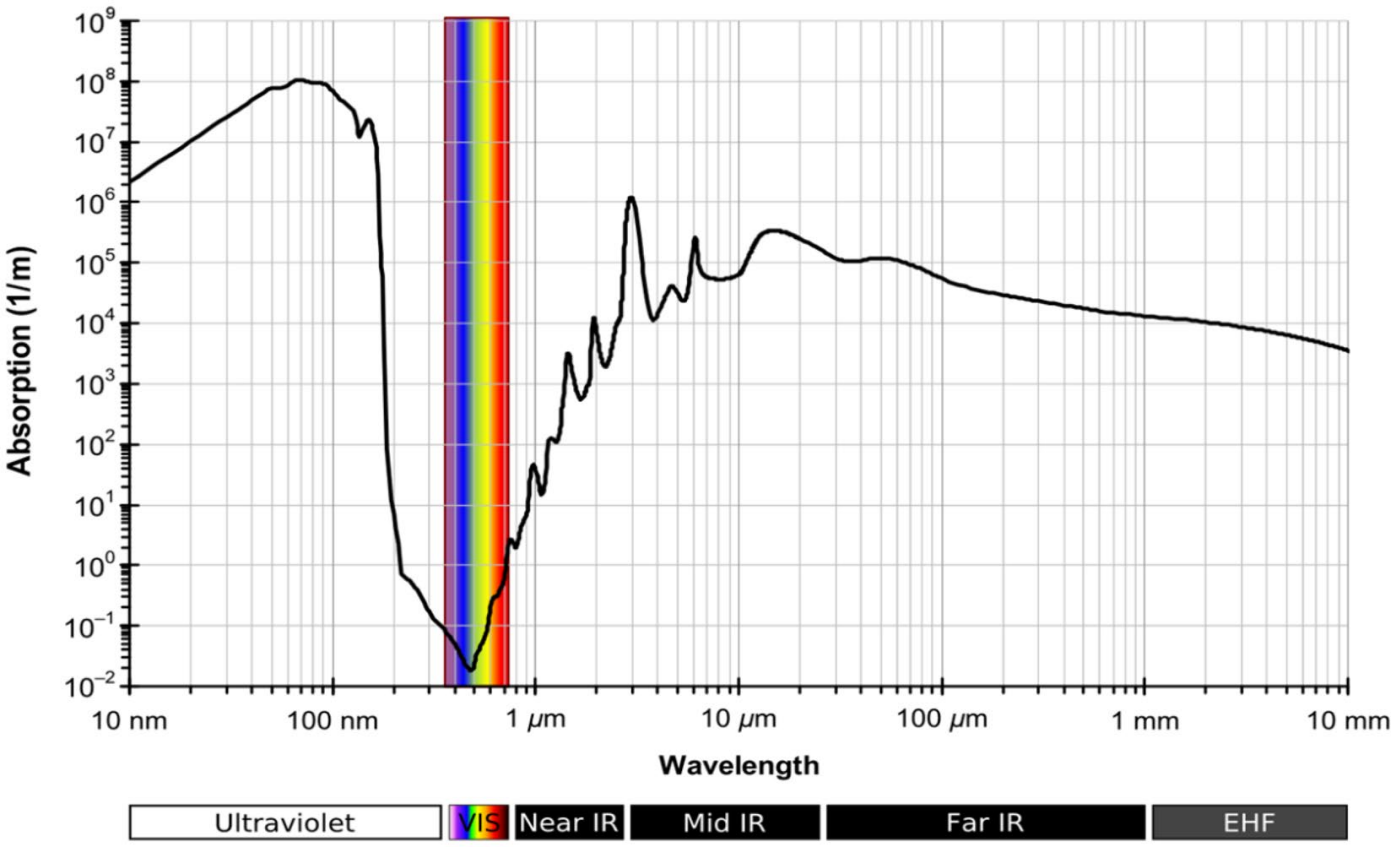
Water absorption spectrum as a function of incident wavelength^36^.

At the end of this subsection, we study the optical properties of water. It is known that the variation of the refractive index of water depends on the incident wavelength, and it varies from 1.15 to 1.5 RIU. As a result, at visible light, the refractive index of fresh water is approximately equal to 1.33 RIU^29^. Moreover, the extension coefficient of water is also varied as a function of the wavelength. Also, we have the absorption spectrum of water as in Fig. 6, which confirms that the water is transparent for the ultra-violet and visible spectrum because of its low absorbance for these wavelengths as shown. In addition, the water is strongly absorbed in the mid and far IR spectrum. Therefore, as we discussed previously in the last section, the saline water index changes from 1.3326 to 1.3505 RIU due to the variation in salt level from 0 to 100%. As a result, we test our structure's ability to characterize the various refractive indexes of saline water as a measure of the salinity level in seawater.

After the optimization of the 2D hexagonal PCs, we became able to localize the PBG as we needed for any specific application. Therefore, in the following subsection, we will study the defected modes of the last structures to be salinity sensors for the saline water in the water desalination techniques.

### Defected two-dimensional photonic crystals

We are concerned about the defective structure for the salinity sensor. Our structure is an infinite crystal made of hexagonal drilled holes within a host material of titanium dioxide $(Ti{O}_{2})$, which we believe to be 2D-PCs as we have shown in Fig. 1. Except for the central hexagonal cylinder, which holds the saline water to be investigated, all the hexagonal cylinders in Fig. 1 are filled with air.

**Figure 7:**
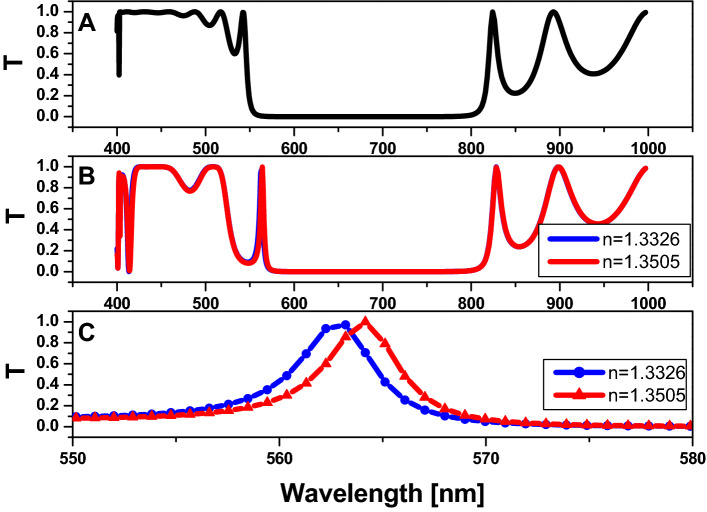
The transmission property of the two-dimensional hexagonal cylinder unit cell with $\mathrm{R}=80\mathrm{ nm}$ $\mathrm{D}=80\mathrm{ nm}$,$\mathrm{N}=5,\mathrm{ and}$ the host material is titanium dioxide $\left({\mathrm{TiO}}_{2}\right)$. (**A**) All cylinders are filled with air$,$ (**B**) All cylinders are filled with air except the centered pour which filled with saline water with different values of refractive index, and (**C**) zoom on defect peak on figure B.

In Fig. 7, the transmission spectrum curve is formed using FEM. In Fig. 7A, this structure consists of the two-dimensional hexagonal cylinder unit cell with $$r=80 nm,$$ $$D=80 nm$$,$$and number of periods (N)=5,$$ at host material made from titanium dioxide $$\left(Ti{O}_{2}\right)$$, and all cylinders are filled with air. As we have shown, there is a PBG formed in the spectral region from 549 to 813 nm. While injecting saline water into the central part of the structure in Fig. 7B, we notice a defect peak at 565 nm. However, we are unable to differentiate between the different refractive indexes of saline water, so we zoom in on the wavelength range from 550 to 580 nm as shown in Fig. 7C. As a result, the attributes of this sensor must be calculated: sensitivity $$(S)=67 nm/RIU$$, $$Q=128 ,$$ and figure of merit $$\left(FOM\right)=15 {RIU}^{-1}$$.

**Figure 8:**
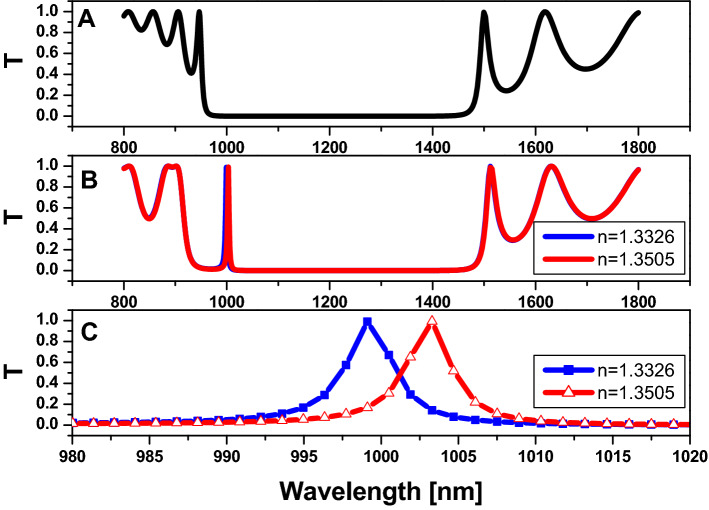
Transmission property of a two dimensional hexagonal cylinder unit cell with $\mathrm{R}=180\mathrm{ nm}$ $\mathrm{D}=120\mathrm{ nm}$,$\mathrm{N}=5,\mathrm{ and}$ the host material titanium dioxide $\left({\mathrm{TiO}}_{2}\right)$. (**A**) All cylinders are filled with air; (**B**) All cylinders are filled with air except for the centered pour, which is filled with saline water with varying refractive index values; and (**C**) zoom in on the defect peak in figure (**B**).

Then, by increasing the dimension of the structure wherein,$R=180 nm$*, *$D=120 nm$*,*$N=5,\mathrm{ and}$ the host material is titanium dioxide $\left(Ti{O}_{2}\right)$*,* all cylinders are filled with air. As we have shown in Fig. 8A, there is a PBG formed in the spectral region from 922 to 1482 nm. While injecting saline water into the central part of the structure in Fig. 8B, we notice a defect peak at 1000 nm. However, we are unable to differentiate between the different refractive indexes of saline water, so we zoom in on the wavelength range from 980 to 1020 nm as shown in Fig. 8C. As a result, the attributes of this sensor must be calculated, sensitivity $(S)=200 nm/RIU$, $Q=285.4 ,$ and figure of merit $\left(FOM\right)=57 {RIU}^{-1}$.

By the same manner, we are unable to differentiate between the different refractive indexes of saline water, so we zoom in on the wavelength range from 550 to 580 nm as shown in Fig. 7C. As a result, the attributes of this sensor must be calculated: sensitivity $(S)=67 nm/RIU$, $Q=128 ,$ and figure of merit $\left(FOM\right)=15 {RIU}^{-1}$.

**Figure 9:**
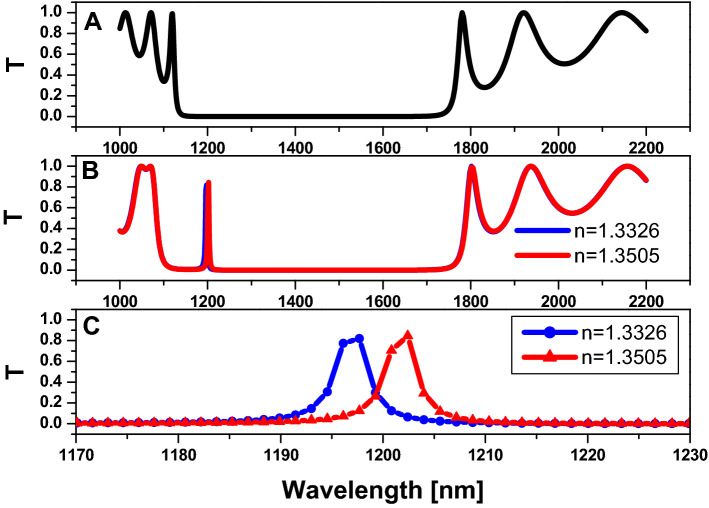
Transmission property of two-dimensional hexagonal cylinder unit cell with $\mathrm{R}=250\mathrm{ nm}$ $\mathrm{D}=120\mathrm{ nm}$,$\mathrm{N}=5,\mathrm{ and}$ the host material is titanium dioxide $\left({\mathrm{TiO}}_{2}\right)$. (**A**) All cylinders are filled with air; (**B**) All cylinders are filled with air except for the centred pour, which is filled with saline water with varying refractive index values; and (**C**) zoom in on the defect peak in figure (**B**).

**Figure 10:**
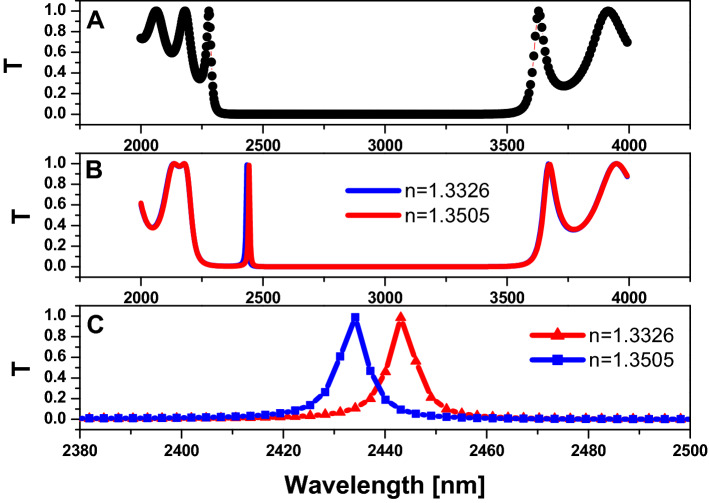
Transmission property of two-dimensional hexagonal cylinder unit cell with $\mathrm{R}=500\mathrm{ nm}$ $\mathrm{D}=250\mathrm{ nm}$,$\mathrm{N}=5,\mathrm{ and}$ the host material is titanium dioxide $\left({\mathrm{TiO}}_{2}\right)$. (**A**) All cylinders are filled with air; (**B**) All cylinders are filled with air except for the centred pour, which is filled with saline water with varying refractive index values; and (**C**) zoom in on the defect peak in figure (**B**).

By the same procedure of increasing the dimension of the structure, wherein, $R=250 nm,$ and $D=120 nm$*,* as in Fig. 9, the sensitivity $(S)=279 nm/RIU$, $Q=324 ,$ and figure of merit $\left(FOM\right)=75 {RIU}^{-1}$. Also, for $R=500 nm,$ and $D=250 nm$*,* as in Fig. 10*,* the sensitivity $(S)=525 nm/RIU$, $Q=376 ,$ and figure of merit $\left(FOM\right)=80.7 {RIU}^{-1}$. So we have an enhancement to the sensing performance by increasing the dimension of the considered structure.

**Figure 11:**
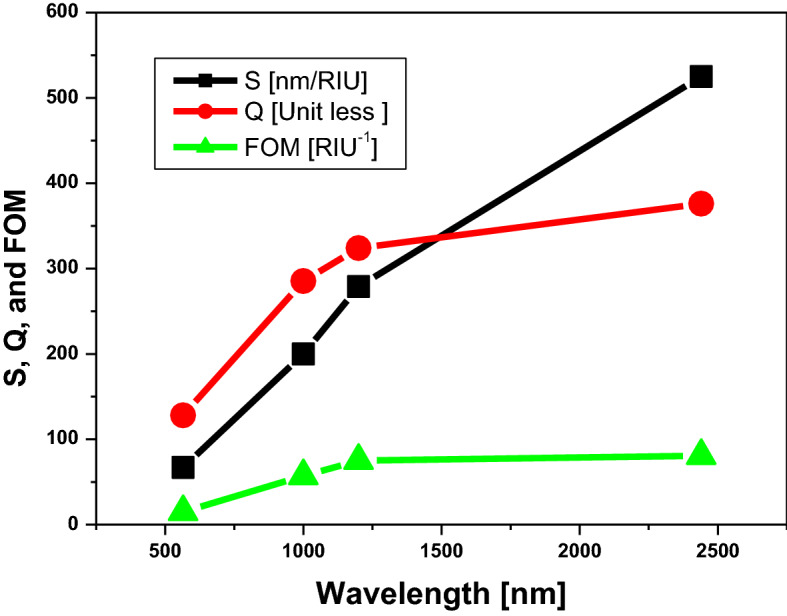
The dependence of sensor parameters on the position of the defect peak.

Table 2 is show that the sensor performance depends on the dimension of the structure, which we discussed previously in this part. Therefore, we plot the relationship between the sensor parameters [S, Q, and FOM] and the position of the defect peak in the PBG region as shown in Fig. 11. We noticed that the sensitivity is increased by shifting the defect peak towards longer wavelengths (Mid-IR spectrum). In addition, as shown in Fig. 11, the figure of merit (FOM) and quality factor (Q) appear to be constant in the longer wavelengths. Thus, the sensitivity of saline water is reached to $525 nm/RIU$ at the defect peak located at the mid IR spectrum owing to the high absorbance of water at the mid IR spectrum, as we discussed previously in Fig. 6. Therefore, our structure of hexagonal cylinder drilled in host materials from titanium dioxide has a high ability to determine the refractive index of saline water which is referred to as the level of salinity.

**Table 2:** The sensor performance of different structures as shown.

Figure	Photonic band gap		Position of defect peak	Sensitivity $(\frac{\mathrm{nm}}{\mathrm{RIU}})$	Quality factor	Figure of merit ${(\mathrm{RIU}}^{-1})$
	From [nm]	To [nm]				
Figure 7	549	813	565	67	128	15
Figure 8	922	1482	1000	200	285.4	57
Figure 9	1129	1748	1200	279	324	75
Figure 10	2304	3566	2440	525	376	80.7

Here, this considered structure from 2D-PCs hexagonal unit cell is an extension to our previous work for the 2D-PCs with a circular unit cell which published at material science and engineering B^31^. The sensitivity of the circular unit cell is reached to 58 [nm/RIU] as published in^37,38^.Thus, the comparison between the circular and hexagonal salinity sensors confirms that the hexagonal shape is high sensitivity for the water salinity rather than the conventional structure of circular unit cell.

## Conclusion

In this paper, we show how to use two-dimensional hexagonal cylinder photonic crystals as a salinity sensor. We design the structures in such a way that all hexagonal cylinders of radius r are filled with air, with the exception of the centred pour, which is filled with saline water with varying refractive index values [1.3326–1.3505 RIU]. We optimized the two-dimensional hexagonal photonic crystals to localize the photonic bandgap region in the mid-infra-red frequency range as water is a good absorber for this range of frequencies. By adjusting the *dimension* of the senso*r* are: $R=500 \mathrm{nm},$ and $D=250 \mathrm{nm}$ to give a PBG from 2304 to 3566 nm, also, the sensitivity $(S)=525 nm/RIU$, $Q=376 ,$ and figure of merit $\left(FOM\right)=80.7 {RIU}^{-1}$. We have an enhancement to the sensing performance by increasing the dimension of the considered structure. The sensitivity changed from 67 $\mathrm{nm}/\mathrm{RIU}$ (PBG in the visible spectrum) to 525 $\mathrm{nm}/\mathrm{RIU}$ (PBG at mid-IR spectrum). Therefore, by increasing the dimensions of the structure such as R and D, the photonic band gap is shifted to longer wavelengths and the sensitivity of the sensor is increased. The COMSOL multiphysics program's finite element method (FEM) is used in our modelling and simulation procedures. It has been proved that the present design can determine the saline water refractive index that corresponds to the salinity level needed to conduct the desalination process. In addition, the recommended device attributes in the field of photosensitive applications were highlighted by these results.

## Data Availability

The datasets used and/or analyzed during the current study are available from the corresponding author on reasonable request.

## Code availability

No code is used in this study.
